# 2036 Extracellular matrix as a novel approach to glioma therapy

**DOI:** 10.1017/cts.2018.73

**Published:** 2018-11-21

**Authors:** Mark H. Murdock, Jordan T. Chang, George S. Hussey, Nduka M. Amankulor, Johnathan A. Engh, Stephen F. Badylak

**Affiliations:** 1 McGowan Institute for Regenerative Medicine, University of Pittsburgh; 2 Department of Neurological Surgery, University of Pittsburgh

## Abstract

OBJECTIVES/SPECIFIC AIMS: Gliomas are the most lethal and common primary tumor type in the central nervous system across all age groups; affected adults have a life expectancy of just 14 months. As glioma cells invade the surrounding normal parenchyma they remodel the composition and ultrastructure of the surrounding extracellular matrix (ECM), suggesting that the native (i.e., “normal”) microenvironment is not ideal for their survival and proliferation. Recent reports describe suppressive and/or lethal effects of mammalian ECM hydrogels derived from normal (nonneoplastic) sources upon various cancer types. ECM-based bioscaffolds placed at sites of neoplastic tissue resection in humans have never been reported to facilitate cancer recurrence. The objective of the present research is to evaluate mammalian ECM as a novel approach to glioma therapy. METHODS/STUDY POPULATION: ECM hydrogels from porcine dermis, small intestine, and urinary bladder were produced as described previously. Primary glioma cells were graciously supplied by Drs. Nduka Amankulor and Johnathan Engh, and U-87 MG were ordered through ATCC. Cells were plated onto tissue culture plastic at ~60% confluence and allowed to attach for 24 hours before treatment. The saline-soluble fraction (SSF) of ECM was obtained by mixing lyophilized, comminuted ECM with 0.9% saline for 24 hours then filtering the resulting mixture through a 10 kDa molecular weight cutoff column. All assays and kits were followed according to the manufacturer’s instructions. Cell viability was measured via MTT assay (Vybrant^®^ MTT Cell Proliferation Assay, Invitrogen) and by live/dead staining (LIVE/DEAD^®^ Cell Imaging Kit, Invitrogen). Time lapse videos were created by taking images every 20 minutes for 18 hours (phase-contrast) or every 10 minutes for 12 hours (darkfield). NucView reagent was ordered from Biotium. Temozolomide was ordered through Abmole. All in vivo work was conducted according to protocols approved by the University of Pittsburgh’s IACUC office. RESULTS/ANTICIPATED RESULTS: ECM hydrogels derived from porcine dermis, small intestine, or urinary bladder all decreased the viability of primary glioma cells in vitro, with urinary bladder extracellular matrix (UBM) having the most dramatic effects. The SSF of UBM (UBM-SSF), devoid of the fibrillar, macromolecular components of ECM, was sufficient to recapitulate this detrimental effect upon neoplastic cells in vitro and was used for the remainder of the experiments described herein. In a cell viability assay normalized to the media treatment, non-neoplastic CHME5 and N1E-115 cells scored 103% and 114% after 48 hours when treated with UBM-SSF and 2 primary high-grade glioma cell types scored 17% and 30.5% with UBM-SSF (n=2). Phase-contrast time-lapse video showed CHME5 and HFF thriving in the presence of UBM-SSF for 18 hours while most primary glioma cells shriveled and died within this time. Darkfield time-lapse video of wells containing Nucview dye, fluorescent upon cleavage by active caspase-3, confirmed that within 12 hours most primary glioma cells underwent apoptosis while CHME5 and HFF did not. In culture with primary astrocytes, high grade primary glioma cells, and U-87 MG glioma cells for 24 hours, UBM-SSF was found to significantly increase the population of primary astrocytes compared with media (*p*<0.05) while decreasing the 2 glioma cell types to approximately one-third as many cells as the media control (*p*<0.0001). A dose-response of temozolomide from 0 to 10,000 μM showed that when treating 2 non-neoplastic cell types (CHME5 and HFF) and 2 types of primary glioma cell there was no difference in survivability at any concentration. Contrasted to this, a dose-response of UBM-SSF from 350 to 7000 μg/mL showed that the non-neoplastic cells survived significantly better than the glioma cells at concentrations of 875 μg/mL and upward (*p*<0.05). In preliminary animal experiments, large primary glioma tumors in the flanks of athymic nude mice were resected and replaced with either UBM SSF or Matrigel (an ECM product of neoplastic cell origin). After 7 days the resection sites with UBM-SSF had little tumor regrowth if any compared with the dramatic recurrence seen in the Matrigel injection sites (n=2). In a separate survival study comparing PBS to UBM-SSF injections in the flank-resection model, all animals given PBS had to be sacrificed at 9, 11, and 11 days (n=3) whereas animals given UBM-SSF were sacrificed at 15, 24, and 39 days (n=3), indicating a moderate increase in survival due to the UBM-SSF. DISCUSSION/SIGNIFICANCE OF IMPACT: Since the introduction of the pan-cytotoxic chemotherapeutic agent TMZ in 2005, the standard of care for patients with glioblastoma multiforme has not improved. These findings indicate that non-neoplastic ECM contains potent bioactive regulators capable of abrogating malignancy. Our in vitro data suggest these molecules appear to have no deleterious effect on non-neoplastic cells while specifically inducing apoptosis in glioma cells. Our in vivo data suggest that these molecules may be useful in delaying glioma recurrence, thus resulting in extended lifespan. Delivering soluble fractions of ECM to a tumor site may represent a novel approach to glioma therapy, sidestepping traditional cytotoxic therapies in favor of utilizing putative endogenous anti-tumor pathways.

